# LoRa-Based IoT Multi-Hop Architecture for Smart Vineyard Monitoring: Simulation Framework and System Design

**DOI:** 10.3390/s26041112

**Published:** 2026-02-09

**Authors:** Chiara Suraci, Pietro Zema, Giuseppe Marrara, Angelo Tropeano, Alessandro Campolo, Mariateresa Russo, Giuseppe Araniti

**Affiliations:** 1 Department of Information Engineering, Infrastructure and Sustainable Energy (DIIES), University Mediterranea of Reggio Calabria, 89124 Reggio Calabria, Italy; pietro.zema@unirc.it (P.Z.); giuseppe.marrara@unirc.it (G.M.); angelo.tropeano@unirc.it (A.T.); cmplsn03d04h224v@studenti.unirc.it (A.C.); araniti@unirc.it (G.A.); 2 Food Chemistry, Safety and Sensoromic Laboratory, University Mediterranea of Reggio Calabria, 89124 Reggio Calabria, Italy; mariateresa.russo@unirc.it

**Keywords:** IoT, D2D, smart agriculture, multi-hop, AI

## Abstract

The growing interest in precision agriculture has led, in recent years, to an increase in the adoption of Internet of Things (IoT) technologies in the service of smart agriculture to optimize agricultural production processes through the monitoring of environmental conditions and prevent food loss. This work stems from research conducted as part of the Tech4You project, where the enabling digital technologies developed in Spoke 6 contribute to the advanced solutions envisaged by Spoke 3 to facilitate the transition to a sustainable agrifood system. In particular, we present the design and evaluation of a multi-hop Device-to-Device (D2D) communication architecture that leverages Long Range (LoRa) technology, specifically designed for monitoring vineyards in the context of passito wine production. The proposed framework addresses the challenge of monitoring mobile containers for grapes during the drying phase, a critical stage in which inadequate temperatures and humidity can promote the growth of fungi and the formation of mycotoxins. The integration of simulation-based performance evaluation with a multi-layer system architecture is presented in this work. The objective is to compare the performance of different routing strategies in choosing data forwarding paths to the gateway. The simulation results show that the proposed routing strategy, which is based on learning but also focuses on energy consumption, offers good performance. In particular, it achieves packet delivery rates of over 92% and preserves over 95% of active nodes after 2 h of operation. Energy-aware routing strategies also perform well compared to those that only consider the distance from the destination, but overall, the proposed strategy achieves a better trade-off on the metrics analyzed.

## 1. Introduction

The rapid proliferation of the Internet of Things (IoT) paradigm has led to the deployment of billions of connected devices for use in various fields, including smart agriculture, industrial automation, environmental monitoring, and logistics. These applications often require energy-efficient, scalable, and resilient communication solutions that can support a large number of heterogeneous devices under varying conditions. Traditional infrastructure-based networks, where all communication flows are relayed through centralized gateways or access points, face significant challenges in such scenarios. Limited coverage, single points of failure, and energy constraints make them inadequate for wide-area and resource-constrained deployments.

Device-to-Device (D2D) communication is a useful tool in this regard, as it allows for direct data exchange between IoT nodes. This paradigm reduces latency, improves energy efficiency, and offloads traffic from central infrastructure, making it highly relevant for dense or infrastructure-poor environments [1]. D2D has already proven effective in supporting localized communication in wireless networks, and is increasingly recognized as the cornerstone of next-generation communication systems [2]. Within fifth-generation (5G) and beyond-5G (B5G) architectures, D2D is closely aligned with massive Machine-Type Communication (mMTC) and Ultra-Reliable Low-Latency Communication (URLLC) service classes, contributing to network densification and edge intelligence.

Among the available technologies, Low-Power Wide-Area Networks (LPWANs), such as SigFox [3], Narrowband IoT (NB-IoT) [4], and Long Range (LoRa)/LoRa Wide Area Network (LoRaWAN) [5], have emerged as promising candidates for long-range, low-energy IoT connectivity [6]. In particular, LoRaWAN has gained popularity due to its open deployment model, low-cost infrastructure, and adaptability to rural [7] and industrial environments [8]. However, a key limitation of LoRaWAN lies in its star-of-stars architecture, which does not natively support multi-hop communication. As a result, end devices located beyond the coverage of a gateway, or those affected by obstacles and interference, may be excluded from the network or forced to increase transmission power, thus reducing the battery life.

Recent research has investigated multi-hop extensions and relay mechanisms to address this limitation. By enabling intermediate nodes to forward packets to the gateway, multi-hop architectures can extend coverage, improve resilience, and balance energy consumption across the network [9]. However, these approaches raise new challenges, including energy asymmetry, where relay nodes deplete their batteries faster than others, increased latency, as additional hops accumulate forwarding delays, and protocol inefficiencies, since LoRaWAN’s ALOHA-based medium access control (MAC) is not optimized for multi-hop operation [10].

To overcome these limitations, this paper proposes a multi-hop D2D architecture that exploits LoRa communications to send data between sensor-equipped containers used in a vineyard to collect critical parameters such as temperature and humidity. This work comes from research conducted as part of the Tech4You (https://ecs-tech4you.it/, accessed on 15 December 2025) project and, in particular, Spoke 6, whose main goal is to develop enabling digital solutions for the creation of new paradigms in the thematic spokes. In this case, the proposed solutions are aimed at Spoke 3, which focuses on advanced digital technologies for sustainable food supply chains. Three routing algorithms are run on the proposed architecture to compare the effects of different traffic management strategies on the scenario under consideration. Two of these, specifically developed for the proposed architecture, are inspired by well-known routing algorithms, favoring the path that guarantees the fewest number of nodes (shortest-path) or the path that minimizes energy consumption (energy-aware). The third is an original proposal in this paper, as it uses Artificial Intelligence (AI) techniques to dynamically select the best routing paths based on both the current battery charge level of the sensor devices and the connection quality and network topology. Instead of relying on static or heuristic routing rules, the algorithm learns and adapts to environmental conditions, ensuring balanced energy consumption among nodes while maintaining reliable data transmission to the base station. In particular, the article aims to demonstrate that, in agricultural and industrial contexts, where devices are often battery-powered, geographically dispersed and used in harsh conditions, intelligent routing could be the key to extending the lifetime of the network and ensuring service continuity [9,11].

The threefold contribution of this paper is articulated through the following points:

1. The design of a LoRaWAN-based multi-hop D2D communication framework tailored for smart vineyard monitoring, addressing the challenges posed by mobility, uneven terrain, and energy-constrained sensor nodes involved in the grape drying process for vino passito production.
2. The development of a realistic simulation environment that integrates a digital elevation model (DEM), terrain-sensitive propagation effects, and testbed-based energy profiling to evaluate large-scale implementations in vineyards.
3. The proposal and evaluation of an adaptive routing strategy, inspired by artificial intelligence, that enables energy-balanced forwarding decisions and comparison with shortest path and energy-sensitive routing policies.

The remainder of the paper is organized as follows. Section 2 provides an overview of the technologies that make D2D possible, with a view to highlight which technology makes D2D most suitable for IoT scenarios. Section 3 discusses the effects of using LoRaWAN-enabled multi-hop D2D communications on selected application scenarios. Section 4 describes the proposed architecture for smart vineyard monitoring. Section 5 presents the experimental results obtained, prefaced by the simulation setup, routing strategies tested, and metrics considered. Section 6 provides an in-depth discussion of the simulation framework for multi-hop models, emphasizing the rationale behind integrating intelligent routing mechanisms. Conclusions are exposed in Section 7.

## 2. Device-to-Device Communication for IoT

D2D communication enables direct data exchange between nearby devices, reducing latency and energy consumption, and offloading traffic from centralized infrastructure. The features of D2D make it particularly well suited to meeting the stringent requirements of IoT environments in terms of scalability, energy autonomy, and flexibility.

Multiple short- and long-range technologies support D2D capabilities with different trade-offs in range, power consumption, throughput, and network complexity. Short-range solutions (i.e., Wi-Fi Direct, Zigbee, Bluetooth Low Energy (BLE), Ultra-Wideband (UWB)) provide localized, low-latency communication and mesh capabilities, while LPWANs such as SigFox, NB-IoT, and LoRaWAN extend connectivity over several kilometers with minimal power usage. Table 1 summarizes the main characteristics of the different technologies that can be leveraged by D2D communications.

LoRaWAN stands out as the most suitable technology for large-scale rural deployments thanks to its long-range coverage, low energy profile, and flexibility for private, operator-free installations. Moreover, emerging multi-hop and relay extensions make LoRa particularly promising for monitoring in complex agricultural landscapes, where obstacles, irregular row geometry, and mobility limit single-hop connectivity.

## 3. Related Works

The adoption of D2D and LPWANs, particularly LoRa/LoRaWAN, has been extensively investigated in the literature across several vertical domains. This section reviews and discusses representative related works grouped by application scenario with the aim of highlighting how multi-hop mechanisms can be employed to enhance coverage, resilience, and energy efficiency. In particular, it focuses on smart agriculture, industrial automation, and logistics, which represent three relevant verticals for LoRaWAN-based IoT systems. By comparing existing solutions in these areas, this section strives to motivate the architectural choices and adaptive routing strategies proposed in this work. Furthermore, the section reviews works in the literature on multi-hop wireless mesh network that, although developed under different scenarios, provide relevant insights into scalable and reliable multi-hop infrastructures.

### 3.1. Smart Agriculture

Agriculture represents a key domain for the deployment of energy-constrained IoT systems. Precision farming relies on continuous monitoring of environmental parameters such as soil moisture, canopy humidity, temperature, and solar radiation. LoRaWAN is particularly suited for such applications due to its long-range communication, low deployment cost, and open network model, as widely discussed in recent works on wireless communication protocols for smart agriculture [12,13]. However, vineyards, orchards, and heterogeneous rural landscapes pose coverage challenges, as obstacles and irregular layouts often limit direct connectivity to gateways. Experimental research on LoRaWAN communications from underground to the surface has demonstrated how propagation conditions and implementation choices significantly influence network performance in agricultural environments [14]. To overcome these limitations, multi-hop extensions of LoRa have been proposed in the literature that allow nodes to forward measurements through neighboring devices. The authors of [15] demonstrate that relaying improves link reliability and reduces the need for high transmission power, thus extending battery lifetime and supporting seasonal autonomy. Applications of multi-hop LoRa in smart agriculture include vineyard monitoring [9], irrigation control [16], and greenhouse automation [17], where energy autonomy and resilience to node failures are critical. Despite the benefits discussed, there are some challenges that need to be addressed, which led us to develop our work in which we propose a multi-hop, energy-aware, and adaptive routing framework, as presented in this article.

### 3.2. Industrial Automation and Smart Factories

In industrial contexts, D2D communication can support machine-to-machine interactions and distributed monitoring of production lines. Short-range technologies such as Zigbee and BLE are widely adopted for local sensing, while LoRa/LoRaWAN enables integration across larger facilities. The combination of these technologies with 5G backhaul provides a hybrid model that leverages local autonomy and global scalability [18,19]. Multi-hop LoRa overlays are particularly beneficial in harsh electromagnetic environments, where metallic structures and machinery obstruct line of sight. Relay nodes improve coverage and fault tolerance, reducing downtime in automated processes [20]. At the same time, edge computing and network slicing in 5G enhance flexibility, enabling real-time analytics for predictive maintenance and adaptive control. Case studies in smart factories confirm that hybrid D2D–LPWAN systems can achieve low latency, scalability, and energy efficiency simultaneously [21].

### 3.3. Logistics and Asset Tracking

Logistics and supply chains increasingly depend on pervasive IoT connectivity to ensure traceability, condition monitoring, and operational efficiency. LoRaWAN has been deployed for asset tracking across warehouses, transport corridors, and cold-chain networks [22,23]. By equipping containers or vehicles with LoRa-based trackers, operators can monitor temperature, humidity, and shocks in real time, preventing losses and ensuring regulatory compliance [24]. Multi-hop configurations further extend coverage in rural or obstructed environments, where gateway density is limited. Hybrid solutions that combine technology as BLE for indoor localization with LoRaWAN for wide-area communication are increasingly adopted [25]. Dashboards and backend systems aggregate sensor data, provide georeferenced visualization, and issue alerts in case of anomalies, thereby improving responsiveness and decision-making. These capabilities are particularly relevant in food supply chains, where compliance with safety standards requires continuous and reliable monitoring [24,26].

### 3.4. Multi-Hop Wireless Mesh Networks

Alongside the analysis of specific LPWAN studies, a significant amount of research has investigated multi-hop wireless mesh networks as scalable and reliable infrastructures for disseminating large amounts of data. For example, the work in [27] focuses on multicast routing and resource-aware strategies in wireless mesh networks aimed at achieving advantages in terms of metrics such as scalability, coverage extension, reliability, and delay control in multi-hop environments. In the context of multi-hop wireless mesh networks, the authors of [28] have studied resource-aware multicast mechanisms to address scalability limitations, interference and throughput degradation along multi-hop wireless paths. They have proposed integrated multicast architectures to improve dissemination efficiency by leveraging multiple access gateways and wired backhaul links as internet shortcuts, thus reducing the effective number of wireless hops. These approaches combine gateway selection strategies based on load and link reliability with interference-controlled multicast tree construction and dynamic group management, thus achieving improved throughput, delay and QoS in large-scale multicast and video broadcast scenarios. Building on these concepts, further studies have extended integrated multicast architectures to multi-source video broadcasting in Internet-connected wireless mesh networks. Shared integrated multicast frameworks have been proposed to support multiple simultaneous video sources by jointly leveraging wired backhaul resources and multi-hop wireless paths. The purpose is to limit control overhead, reduce interference, and balance the utilization of wireless and Internet resources [29].

#### Cross-Sector Summary and Design Motivations

An analysis of the literature reviewed in the previous subsections shows that the performance of LoRaWAN-enabled multi-hop D2D communications in various application environments can be significantly affected by how data forwarding is managed within the network.

In smart agriculture implementations, multi-hop communication is commonly exploited to cope with uneven terrain and vegetation, thus improving coverage and enabling nodes to operate at lower transmission power. As discussed in the analysis of the related literature, this approach has a positive impact on the energy consumption of the network.

In industrial settings, multi-hop forwarding is primarily adopted to increase robustness against link disturbances caused by machinery and metal infrastructure. By providing alternative communication paths, forwarding nodes can contribute to greater fault tolerance and to mitigate disruptions in automated processes.

In logistics and asset tracking applications, forwarding mechanisms are used to maintain connectivity in environments characterized by limited access to the gateway or physical obstacles, enabling data collection along transport routes and within storage areas.

Although the specific requirements for using them vary by field, the reviewed literature consistently identifies coverage extension and scalability as the main reasons for using multi-hop D2D LoRaWAN architectures in large or irregular deployments. This is the reason why the primary purpose of the proposed multi-hop D2D LoRaWAN is to extend network coverage and improve scalability in irregular agricultural deployments, while ensuring energy sustainability and reliable data delivery. Short end-to-end delays are not a primary design objective, but rather, they are implicitly constrained through hop-count-aware routing decisions.

## 4. System Design of the Proposed Multi-Hop D2D Framework

**Figure 1:**
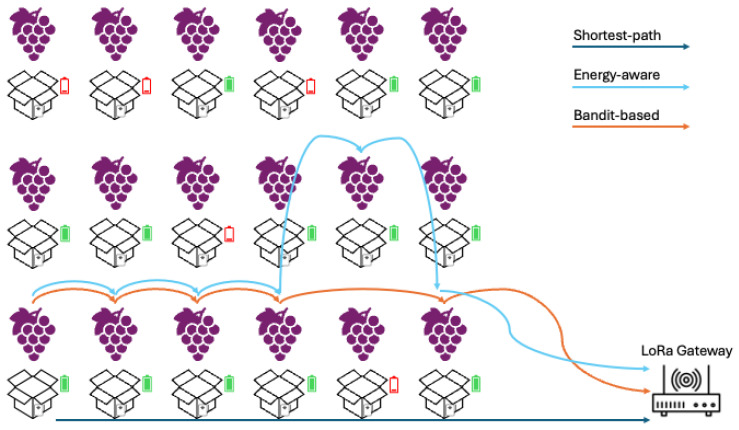
Multi-hop D2D architecture for LoRaWAN-based deployments. The green and red battery icons are useful for showing the impact of device charging on routing decisions.

Among the use cases discussed above, this study focuses on smart agriculture. The reference scenario consists of a vineyard instrumented with a network of sensorized grape containers organized according to the architecture illustrated in Figure 1. The vineyard includes grape containers in mobility that can move between rows and drying areas. There are environmental obstacles in the vineyard, such as vine support structures, metal surfaces, and uneven topography, as these factors are known to affect LoRa propagation. Each grape container is equipped with temperature and humidity sensors, a battery monitoring circuit, and a LoRa transceiver that complies with sub-GHz band regulations. Moreover, Global Navigation Satellite System (GNSS) antennas are installed for localization purposes. Alternative positioning methods, such as Received Signal Strength Indicator (RSSI) triangulation, Time Difference of Arrival (TDOA), and routing-path inference, can be used to assess both high-precision and energy-efficient localization strategies [30].

The purpose of this study is to evaluate the performance of different routing algorithms on a LoRaWAN-based multi-hop D2D architecture specifically designed for vineyard monitoring. In particular, the study aims to validate the effectiveness of multi-hop forwarding in extending vineyard coverage. Traditional LoRaWAN relies on a star topology, which can exclude nodes located beyond the gateway’s range or in obstructed positions. By contrast, multi-hop communication enables packets to traverse multiple intermediate nodes before reaching the gateway, thus extending the operational footprint and improving network resilience.

Unlike generic IoT deployments, our proposal focuses on a highly sensitive agronomic process: the drying phase of grapes for vino passito production. During this stage, unsuitable temperatures or humidity conditions can lead to fungal growth and mycotoxin formation, directly affecting product quality and safety. Consequently, the monitoring infrastructure must ensure continuous sensing of environmental parameters, enable prompt actuation in the event of anomalies, and provide reliable traceability throughout the drying process. The proposed solution is conceived to cope with several system-level requirements that arise in realistic vineyard monitoring deployments. The use of a multi-hop D2D LoRaWAN architecture primarily aims at extending network coverage, allowing sensor nodes placed in unfavorable or distant positions to reach the gateway through intermediate devices. Routing decisions are then exploited to improve reliability, by favoring paths that remain stable under heterogeneous propagation conditions and container mobility. Energy sustainability is a central design concern since the nodes are battery-powered and are expected to operate for long periods without maintenance. For this reason, energy-aware and learning-based routing strategies are adopted to avoid overloading a limited subset of relays and to balance the forwarding effort across the network. Latency is not treated as a dominant constraint in this application, but its impact is implicitly limited by controlling the number of hops along the selected routes. These design choices define a clear correspondence between architectural components, routing strategies, and the performance metrics evaluated in the simulation campaign.

The proposed architecture follows a multilayer design comprising detection, processing, communication, and interface layers, as commonly adopted in IoT and smart agriculture systems [31]. The proposed framework pursues three main functions:

- Periodic collection of environmental parameters, including temperature, humidity, and node battery levels. The latter are particularly useful for dynamically adapting traffic routing to the energy conditions of the devices.
- Detection of mycotoxin contamination risks at the backend level. Risk detection relies on temperature and humidity profiles to classify containers as normal, warning, or critical, triggering a real-time visual alert via orange or red LEDs.
- Container localization and visualization on a georeferenced dashboard. GNSS data are complemented with alternative methods (i.e., RSSI, TDOA, and path inference) to ensure traceability even under energy-constrained conditions.

These functionalities collectively define the operational logic of the proposed framework and motivate the implementation of an adaptive, energy-aware routing mechanism tailored to the vineyard scenario. From a networking perspective, the proposed framework leverages multi-hop D2D communication among LoRaWAN end devices. This allows some nodes to forward data on behalf of others when a direct link to the gateway is unreliable or inefficient. Rather than relying on pre-established routes, each node independently decides whether to forward a packet by considering simple, locally available indicators such as link quality and current battery level. Information about nearby nodes is gathered through uplink transmissions and packet reception without additional signaling. Over time, these local choices create multi-hop paths that reflect the network’s actual operating conditions, including changes in energy availability, node mobility within the vineyard, and propagation effects caused by the environment. In this way, coverage can extend beyond the limits of the traditional LoRaWAN star topology while maintaining low protocol overhead and respecting the strict energy constraints typical of agricultural deployments. Multi-hop forwarding decisions are dynamically adapted based on residual energy and local network conditions, preventing static configurations that could compromise the delivery of the data.

Once packets reach a gateway, data are aggregated and forwarded to the backend system, where environmental monitoring, risk assessment, and georeferenced visualization are performed. Each container transmits temperature and humidity readings, node battery status, and container identification and location metadata. On the backend side, this information is aggregated into a time-series database tracking the lifecycle of each container, from filling to emptying. Lightweight protocols such as MQTT or Hypertext Transfer Protocol (HTTP)/Representational State Transfer (REST) are used for integrating between gateways and backend servers, ensuring interoperability and scalability of the system.

This holistic design is motivated by the need to validate the proposed framework against real deployment requirements, including regulatory duty-cycle constraints, heterogeneous terrain, and seasonal autonomy of battery-powered devices [32].

## 5. Simulation Campaign

**Figure 2:**
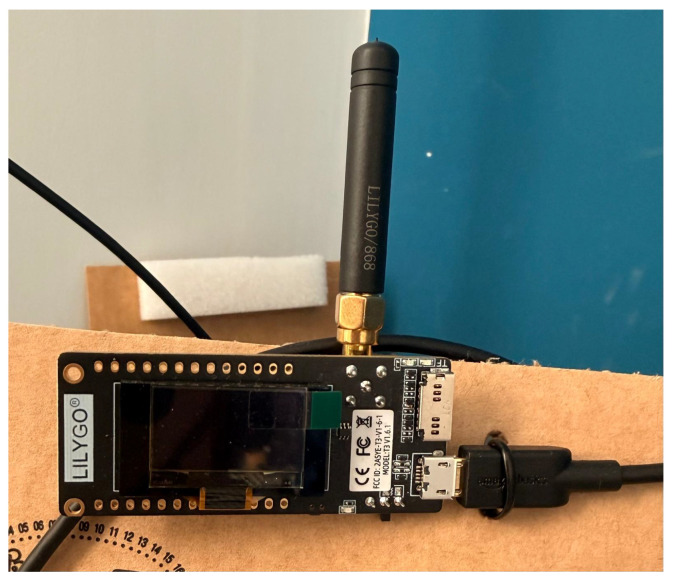
LilyGO T3 v1.6.1 device used as representative of low-power LoRa-enabled microcontroller.

To test the proposed framework by applying the different routing strategies, a comprehensive simulation campaign was conducted under realistic implementation conditions. To accurately model the energy behavior of the sensor nodes, preliminary measurements were performed using LilyGO T3 v1.6.1 devices (by LILYGO, Shenzhen, China), as illustrated in Figure 2. These devices are representative of low-power LoRa-enabled microcontroller platforms that can be deployed on grape containers to collect environmental parameters and transmit data over LoRa. The energy consumption profiles obtained from the hardware measurements were then used to parameterize a network-level simulator, enabling the evaluation of node behavior in a vineyard-scale deployment that reproduces realistic spatial and operating conditions. The remainder of this section describes the simulation setup, the routing strategies implemented, and the metrics measured, concluding with an analysis of the results obtained.

### 5.1. Simulation Setup

To evaluate the proposed framework under realistic topographic and propagation conditions, we developed a Python-based (Python 3.10) simulation environment that geographically reproduces the structure of a Mediterranean vineyard, based on open geographical data from the Chianti area in central Italy, chosen as a reference scenario because it represents a well-known example of Mediterranean viticulture, characterized by large expanses of vineyards, uneven terrain, and significant variations in altitude. The area was obtained from OpenStreetMap and converted into a spatial domain consisting of several plots, each filled with parallel rows representing the vines, with adjustable spacing between rows and spacing between sensors. For the reference configuration, sensors were placed at every 200 m along the rows, and the distance between rows was set to 40 m, resulting in 2702 nodes distributed over approximately 4.3 km^2^.

A single LoRa sink (gateway) was positioned in the lower corner of the domain, as illustrated in Figure 1, to simulate a realistic deployment in which the gate is installed close to a service road or a utility area. The simulated environment allows alternative sink placements, including central and elevated positions, to analyze the variation in coverage due to topography. Each node generates periodic messages containing temperature, humidity, and battery status at a nominal rate of five packets per hour. To keep the computational cost of the simulations manageable, the duration of each simulation was limited to two hours. This allowed for the averaging of small run-to-run variability, which is mainly associated with traffic timing and adaptive routing decisions, without altering the qualitative comparison between routing strategies.

To ensure realism, the simulation was validated against typical vineyard layouts. Container mobility was modeled according to operational practices, with relocation between drying areas. Obstacles such as vine rows and metallic equipment were included to reproduce the propagation environment. The localization accuracy was tested with multiple methods, showing the feasibility of hybrid solutions that combine GNSS with energy-efficient alternatives.

From a protocol perspective, the simulated system adopts a layered design. At the physical layer, nodes employ sub-GHz LoRa modulation for long-range, low-power communication. The MAC layer is based on LoRaWAN Class A operation, augmented with peer-to-peer multi-hop support to overcome the limitations of the star topology. The radio transmission parameters adopted in the simulations, including bandwidth, coding rate, transmission power, and payload length, are consistent with the hardware configuration used for energy profiling and are described in detail in the following. At the routing layer, the system is modeled according to a lightweight multi-hop approach, inspired by protocols known as Ad hoc On-Demand Distance Vector (AODV) or Dynamic MANET On-demand (DYMO), which are on-demand routing solutions commonly used in multi-hop networks. In this work, these protocols are not implemented in their standard form, but are referred to as conceptual models to describe dynamic routing behavior, suitably adapted to the complexity and energy constraints typical of LoRa networks [33]. Finally, the application layer encapsulates temperature and humidity readings, battery status, and container metadata into each payload, ensuring that both environmental monitoring and energy information are available to the backend.

**Figure 3:**
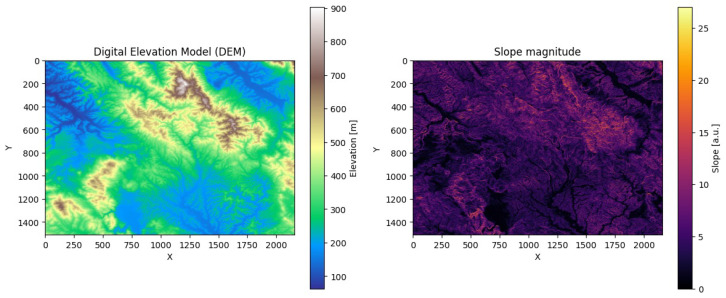
Digital Elevation Model (DEM) of the considered vineyard area and corresponding slope magnitude map.

Furthermore, elevation of the terrain strongly influences long-range communication in rural areas. To capture this effect, a DEM with 30 m (SRTMGL1) was integrated for the simulation campaign carried out. The DEM was projected into the UTM 32N reference system to ensure consistent metric distances and then cropped to the vineyard area, including a 50 m margin beyond the boundaries to avoid edge effects or discontinuities in the calculations. Figure 3 provides an overview of the elevation of the terrain and the slope characteristics of the vineyard area considered in the simulations.

During simulation, each potential link between two nodes was associated with an elevation profile extracted from the DEM. This profile was then processed according to the selected radio propagation model, which determined how terrain elevation affected signal attenuation. Three propagation models with increasing levels of realism were implemented:

- Free-space (FSPL): Purely distance-based, ignoring obstacles. Used for baseline tests and algorithm debugging.
- DEM-aware (line-of-sight): Accounts for the elevation gradient along the link and blocks communication when the terrain obstructs the direct path.
- Knife-edge diffraction: Adds a correction term for partial shadowing, modeling signal bending over ridges. Although computationally heavier, it more accurately represents rural propagation conditions.

For each candidate link, the simulator samples the terrain profile between the two nodes from the DEM and checks the clearance with respect to the straight line of sight connecting them. In the DEM-aware mode, whenever the terrain intrudes into this line, the link is penalized with a fixed non-line-of-sight (NLOS) loss; otherwise, no penalty is applied. In knife-edge mode, the terrain profile is scanned for diffracting ridges, and a diffraction penalty is estimated along the path, with the most obstructing ridge determining the final attenuation. This penalty acts as an additional attenuation that shortens the usable communication range of the link. Instead of computing a full link budget against a sensitivity threshold, the simulator interprets the terrain penalty as a contraction of the nominal range and evaluates feasibility against spreading-factor-specific limits (SF7–SF12), as demonstrated in [34]. The probability of per-hop delivery decreases smoothly as the effective range approaches the threshold, consistent with empirical studies on LoRa reporting a gradual degradation of packet delivery near the link limit [35,36,37].

### 5.2. Compared Routing Policies

In our simulation campaign, we implemented three routing policies that were executed sequentially over the same sensor network topology, ensuring that differences in delivery ratio or latency were due only to routing behavior.

1. Shortest-path: Every node forwards messages through the route with the minimum number of hops to the sink, regardless of energy balance. This method maximizes the delivery rate, but is likely to overload the central relays.
2. Energy-aware: Routes are computed by minimizing the total transmission cost considering both link loss and residual energy. This approach aims to balance the lifetime and reliability of the device.
3. Bandit-based: Each node uses an ε-greedy multi-armed bandit strategy to choose among its feasible neighbors. The rewards depend on the success of the delivery and the local energy state, enabling adaptive learning over time.

Regarding the proposed bandit-based routing policy, each node implements an ε-greedy multi-armed bandit algorithm [38] that enables adaptive next-hop selection without prior knowledge of link quality or energy state. In this formulation, each neighbor candidate for the relay role is treated as an arm associated with an expected reward that reflects both the delivery success and the remaining energy. At each transmission opportunity, the node selects the neighbor with the highest current expected reward (exploitation) with probability 1−ε, or a random neighbor (exploration) with probability ε, where ε∈[0,1] controls the trade-off between exploration and exploitation. After each successful end-to-end delivery, the node updates the expected reward $Q_{i}$ of the selected neighbor *i* using an exponential moving-average rule:

(1) $$Q_{i}\left(t+1\right)=\left(1-\alpha \right)\;Q_{i}\left(t\right)+\alpha \;r_{i}\left(t\right),$$

where α∈[0,1] is the learning rate and $r_{i}\left(t\right)$ is the instantaneous reward reflecting both delivery success and energy expenditure. Specifically, $r_{i}\left(t\right)$ combines a success bonus penalized by the time-on-air and the transmission energy consumed during forwarding, thus balancing reliability and energy efficiency. This moving-average update gives higher weight to recent outcomes while gradually discounting older experiences, enabling each node to adapt its neighbor preferences through ε-greedy exploration and exploitation without requiring global coordination.

### 5.3. Metrics Evaluated

The choice of the evaluation metrics follows the design motivations discussed in the previous sections and reflects the constraints of large-scale vineyard monitoring. Network coverage is not interpreted as a static property, but as a dynamic condition that depends on how long sensor nodes are able to participate in data forwarding before energy depletion occurs. For this reason, the evolution of active nodes over time is used to characterize coverage sustainability. Reliability is evaluated by means of the packet delivery ratio, which provides a direct indication of the effectiveness of the routing strategies in maintaining end-to-end communication under heterogeneous propagation conditions and container mobility. Energy consumption is analyzed by jointly observing node survivability and the average energy cost associated with message forwarding. Latency is not treated as a strict performance requirement in the considered scenario; however, the average hop count is monitored to account for the additional forwarding effort introduced by multi-hop paths and its potential impact on delivery time. The simulation campaign was carried out to evaluate the following metrics:

- Alive nodes represents the number of sensors that retained non-zero battery levels at the end of the simulation, computed as the total number of nodes minus those whose battery charge reached zero.
- Average hop count corresponds to the mean number of forwarding hops required for a message to reach the gateway, calculated as the total number of hops divided by the number of transmission attempts.
- Packet Delivery Ratio (PDR) quantifies network reliability and is computed as the fraction of successfully delivered messages over the total number of attempted transmissions.
- Mean energy value reflects the relative energy consumption per transmitted message. Energy is expressed in normalized battery units (one unit per forwarding event), representing relative consumption rather than absolute Joules, as no hardware-specific power model was included.

As anticipated, realistic energy consumption values were obtained through an experimental testbed designed to model representative LoRa-based sensor node hardware, based on the LilyGO T3 v1.6.1 platform. Specifically, each node was implemented using an ESP32 microcontroller, with an average current draw of approximately 68 mA (Wi-Fi module disabled for field use), and a Semtech SX1276 LoRa transceiver operating at 868 MHz, with a bandwidth of 125 kHz, coding rate 4/5, and spreading factor SF7 or SF8, with the low data rate optimizer disabled. The LoRa frame structure included an 8-symbol preamble, an explicit header disabled, a 5 B-MAC header, a sensor payload of 14 B, and CRC enabled. Under these settings, the time-on-air (ToA) per packet and the corresponding transmission energy were estimated using the official Semtech LoRa calculator tool (https://www.semtech.com/design-support/lora-calculator, accessed on 15 December 2025).

All metrics are evaluated relative to the simulation progress, which represents the fraction of transmitted messages processed over the total number scheduled. This normalized time scale enables direct visual comparison between routing policies despite differences in internal timing or message scheduling.

### 5.4. Experimental Results

**Figure 4:**
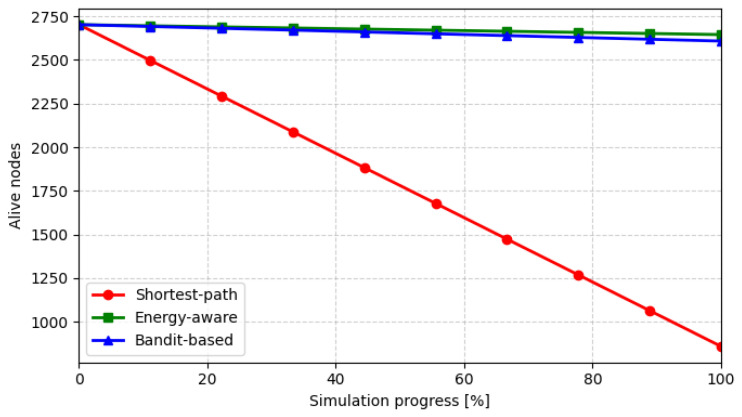
Evolution of active nodes during simulation progress.

Figure 4 illustrates the evolution of active nodes over the 2-h DEM-based simulation run. When the shortest-path policy is applied, energy consumption becomes highly uneven: nodes located along frequently used routes deplete their batteries rapidly, leading to a nearly linear decline in the number of active nodes. In contrast, the energy-aware and bandit-based routing schemes distribute the forwarding load more uniformly across the network, resulting in a slower reduction in active nodes and an extended overall network lifetime.

**Figure 5:**
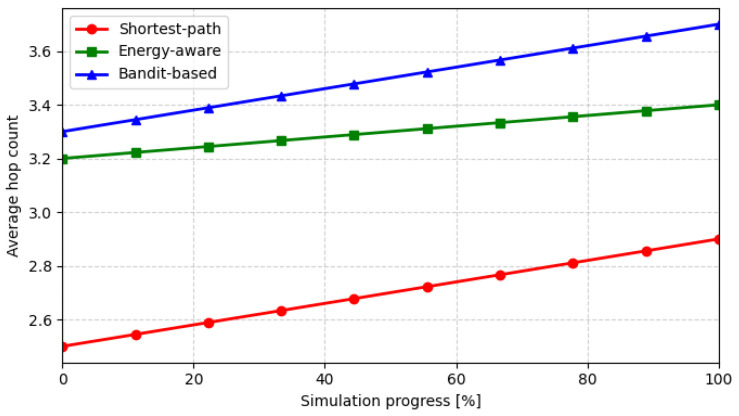
Evolution of the average hop count during simulation progress.

The evolution of the average hop count in Figure 5 provides additional insight into the routing behavior of the three strategies. As expected, the shortest-path policy consistently minimizes the number of hops, but at the expense of a higher energy concentration around central relays, leading to early node depletion (as shown in Figure 4). The energy-aware algorithm introduces slightly longer routes to bypass heavily loaded nodes and to exploit alternative relays located on favorable terrain, resulting in an improved energy distribution across the network. The bandit-based approach exhibits a gradual increase in hop count during the simulation, reflecting its adaptive learning process: as the ε-greedy agent discovers more energy-efficient paths, it occasionally selects suboptimal neighbors to refine its reward model. This dynamic adjustment explains the small but consistent increase in hop count over time and supports the observed balance between network lifetime and delivery reliability.

**Figure 6:**
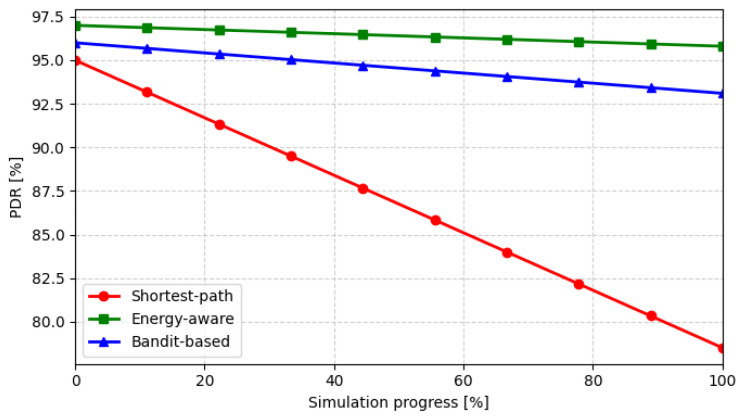
Evolution of the packet delivery ratio (PDR) during the simulation progress.

Figure 6 shows the evolution of the PDR during the simulation progress, highlighting the reliability achieved by each policy. The shortest-path strategy rapidly degrades due to overloaded relays, whereas both energy-aware and bandit-based approaches maintain high delivery ratios throughout the run. The small gap between the latter two confirms that the bandit-based learning mechanism provides reliability comparable to the deterministic energy-aware routing.

**Figure 7:**
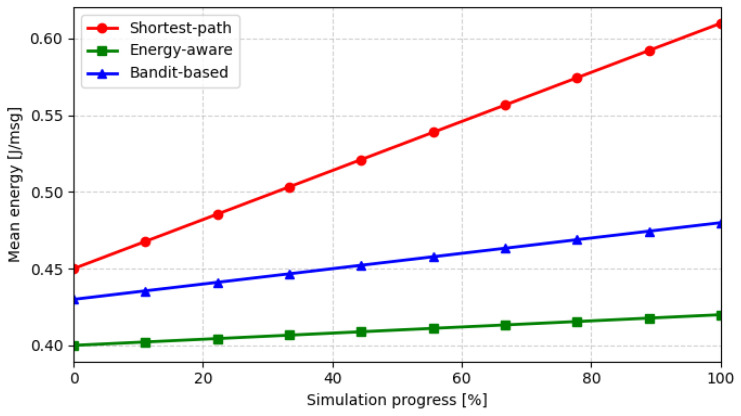
Evolution of mean energy consumption during the simulation progress.

Figure 7 presents the evolution of the mean energy consumption per message transmitted. The energy-aware algorithm achieves the lowest overall expenditure as it explicitly minimizes transmission cost in the routing decision. The bandit-based strategy converges toward a similar energy level after its exploration phase, demonstrating its ability to adaptively learn efficient forwarding paths while preserving delivery performance.

The most significant quantitative results obtained from the simulation campaign are summarized in Table 2, which reports the average value for each metric over a representative two-hour DEM-based simulation involving 2702 sensor nodes. These values represent the system’s intermediate behavior and enable consistent comparative evaluations of the routing strategies. Due to the computational complexity of DEM-based simulations with ground sensing, longer execution times would be required to achieve complete statistical stabilization. However, the presented results already capture the dominant performance trends discussed in this section.

## 6. Discussion

The results confirmed that multi-hop communication provides a tangible improvement in connectivity. Even in irregular layouts, where vine rows and terrain features limit line of sight, multi-hop maintained end-to-end connectivity that would not be possible with single-hop links alone. Coverage remained robust across extended areas, with packet delivery ratios preserved up to several hops. These findings are consistent with recent classifications of multi-hop LoRaWAN approaches that highlight their potential in rural scenarios [39]. However, the simulations also underlined that increased coverage comes at the cost of higher complexity in routing, additional forwarding delays, and greater dependence on intermediate nodes.

### 6.1. The Impact of Routing on Coverage, Reliability, and Energy Sustainability

The evaluation of the proposed framework was conducted focusing on three key metrics that also have an impact on: (i) network coverage, defined as the percentage of nodes that maintain connectivity with the gateway; (ii) reliability, expressed in terms of PDR; (iii) energy consumption, measured between sensor nodes and forwarding nodes to assess network lifetime and balance.

The results relating to alive nodes highlight how routing policies directly influence network coverage, as they impact the spatial and temporal availability of the detection infrastructure. As nodes fail unevenly, connectivity gradually shifts from a global to a fragmented topology, reducing the effective monitoring area. This suggests that maximizing coverage is not only a matter of transmission range but also of energy distribution along forwarding paths. Policies that balance the forwarding task across multiple nodes can preserve connectivity over time, preventing premature isolation of peripheral nodes. In this sense, coverage sustainability becomes an indicator of routing correctness, an essential property for long-term monitoring in sparse or energy-constrained deployments. These observations on coverage are directly related to reliability, as gradual loss of nodes also affects data delivery performance.

Reliability is assessed in relation to the PDR trends presented in the previous section. The vineyard scenario is particularly challenging for reliable communication due to the mobility of containers and interference caused by natural and artificial obstacles. The simulation campaign confirmed that, although multi-hop provides extended coverage, it also introduces the need for trade-offs. Each hop adds a forwarding delay and a potential point of failure. As the number of hops increases, retransmissions may become more frequent and latency would accumulate proportionally to the number of hops, while remaining within thresholds suitable for the context. These results are consistent with previous analyzes showing that LoRaWAN’s ALOHA-based medium access is not optimized for multi-hop environments, which can lead to collisions and inefficiencies [20]. Our work highlights the importance of designing routing strategies that balance connectivity with reliability, ensuring that monitoring objectives are achieved without overloading the network. However, the observed benefits in terms of coverage and reliability can only persist if the underlying forwarding infrastructure remains energy sustainable. This motivates a discussion of the impact of the strategies analyzed on energy consumption, revealing how routing approaches affect the node lifetime and the balance of forwarding workloads.

Energy autonomy is a fundamental requirement in IoT implementations for smart agriculture, as sensor nodes are often left in the field for entire seasons without maintenance. Analysis of energy consumption has shown a clear divergence between routing strategies: the shortest-path approach, while reducing transmission distances, leads to rapid depletion of intermediate nodes, resulting in network fragmentation. Energy-aware and bandit-based strategies, on the other hand, distribute forwarding activity more evenly, resulting in more gradual and balanced consumption. These results confirm that routing decisions directly affect the overall sustainability of the network. In addition to path length, routing policies should focus on the equitable distribution of energy loads to prevent the early exhaustion of a few nodes from compromising long-term coverage and reliability. In this sense, energy balance becomes an essential condition for the scalability of multi-hop LoRa systems in vineyards.

In addition to these performance and sustainability results, it is also important to consider the architectural implications of the proposed approach. From an architectural perspective, the decentralized nature of bandit-based policy inherently supports scalability. Since each node independently learns its forwarding preferences based on local observations, the routing process does not rely on global state or coordination. This allows the network to scale to larger implementations without significantly increasing communication overhead or control complexity, a desirable property for distributed IoT scenarios such as vineyards.

### 6.2. System-Level Implications and Deployment Considerations

Overall, the results obtained through the experimental activities confirm that LoRaWAN-based multi-hop D2D communication is a viable solution for extending connectivity in irregular vineyard layouts, on condition that routing decisions explicitly account for energy sustainability and environmental constraints. From a network design perspective, these findings motivate a multilayer architecture encompassing clearly separated sensing, processing, communication, and interface layers. Functional responsibilities are distributed among nodes based on operational needs, distinguishing devices primarily dedicated to sensing from forwarding nodes and gateways, which aggregate data and provide backhaul connectivity. This solution is flexible and adaptable to different network conditions, thereby reducing excessively asymmetrical energy consumption.

The impact of terrain further reinforces the need for such adaptive designs. The comparison between free-space and DEM-aware configurations highlighted the significant impact of topography on link feasibility. In the free-space baseline, all nodes were nominally connected with an average hop count below two. When DEM-based losses, including knife-edge diffraction, were applied, several links exceeded the receiver sensitivity threshold, leading to partial disconnections in low-visibility areas such as valleys and behind ridges. These effects cannot be compensated by routing alone and must be considered during network planning and deployment.

From a system perspective, the backend mainly addresses the scalability and usability issues that emerged during the simulation analysis. Rather than acting only as a data sink, it combines environmental monitoring, alert handling, and georeferenced visualization, making it easier to relate sensing data to network conditions. In practice, this allows operators to identify problematic areas in the vineyard, for example, where additional gateways, a denser node deployment, or alternative forwarding paths may be required.

The results also make it clear that terrain and energy constraints cannot be treated as secondary aspects in multi-hop LoRa deployments. DEM-based simulations were particularly useful in identifying shadowed regions and candidate forwarding locations, as well as in exposing the trade-offs between hop count, latency, and energy consumption. Future work will focus on extending the model with stochastic channel effects and longer observation periods, in order to verify the stability of adaptive routing strategies under changing environmental conditions and to move toward controlled field trials and digital twin integration.

## 7. Conclusions

In this work, we presented a LoRaWAN-based multi-hop architecture tailored for smart vineyard monitoring, with a focus on the drying process for the production of passito wine. Through a simulation campaign capable of reproducing realistic conditions that take into account the terrain and the actual energy consumption of the devices used, we have demonstrated that multi-hop D2D communication can significantly extend coverage in rural implementations, while preserving delivery reliability and energy autonomy. The proposed evaluation is based on simulations of limited duration and focuses on comparative performance trends rather than on absolute network lifetime estimation. Nevertheless, the performance metrics considered capture the cumulative effects related to energy consumption, forwarding load distribution, and connectivity degradation, which already emerge in the early stages of network operation. Integration of AI-based, energy-sensitive, and learning-based routing strategies mitigates energy asymmetry and improves network sustainability, two key requirements for long-term agricultural monitoring. As these effects are driven by the intrinsic properties of routing strategies, the observed comparative trends are expected to persist over longer operational periods. At the same time, extended simulation horizons and experimental validation would be required to fully capture long-term dynamics, environmental variability, and deployment-specific effects. The proposed framework therefore lays the foundation for future work focusing on predictive analytics, anomaly detection, and digital twin platforms for food supply chain optimization, as well as small-scale laboratory and field experiments aimed at validating the simulation results under real deployment conditions.

## Figures and Tables

**Table 1:** Comparison of D2D enabling technologies.

Technology	Range	Power Consumption	Key Features	Limitations
Wi-Fi Direct	up to 200 m	Medium–High	High throughput, flexible P2P formation	Energy demanding, limited suitability for battery-powered IoT
Zigbee	10–100 m	Very Low	Low-energy mesh, good for dense indoor sensing	Short range, interference in 2.4 GHz band
BLE	10–100 m	Low	Robust mesh networking, industrial adoption	Limited throughput, short range
UWB	10–30 m	Medium	High-precision localization (cm-level), low latency	Short range, higher energy vs BLE/Zigbee
SigFox	up to 50 km	Very Low	Ultra-narrowband, multi-year battery life	Small payloads, restricted bidirectionality, operator-dependent
NB-IoT	1–10 km	Medium	Licensed spectrum, secure two-way communication	Higher energy consumption, operator-managed
LoRa/LoRaWAN	2–15 km	Very Low	Long range, low cost, private networks, multi-hop extensions possible	Star topology by default, low throughput

**Table 2:** Summary of the quantitative results for each evaluated metric (DEM-based simulation, 2 h, 2702 nodes).

Policy	Alive Nodes	Avg Hop Count	PDR [%]	Mean Energy [J/msg]
Shortest-path	860	2.9	78.5	0.61
Energy-aware	2645	3.4	95.8	0.42
Bandit-based	2608	3.7	93.1	0.48

## Data Availability

Data is contained within the article.
